# Supplementary material for: Probabilistic framework for integration of mass spectrum and retention time information in small molecule identification
Source: Bioinformatics. 2020 Nov 27;37(12):1724–31. doi: 10.1093/bioinformatics/btaa998 (PMC8289373; doi:10.1093/bioinformatics/btaa998)
Supplement: btaa998_Supplementary_Data [file btaa998_supplementary_data.pdf]

Probabilistic Framework for Integration of Mass Spectrum and Retention Time Information in Small Molecule Identification

Eric Bach [1,*], Simon Rogers [2], John Williamson [2] and Juho Rousu [1]

[1]Department of Computer Science, School of Science, Aalto University, Espoo, Finland and

[2]School of Computing Science, University of Glasgow, Glasgow, United Kingdom.

* To whom correspondence should be addressed.

Contents

S.1 Code and Data links 1

S.2 Hyper-parameter Estimation 1

S.3 Results 2

S.3.1 Comparison of the Edge Potential Functions 2

S.3.2 Retention Order Prediction Model 2

S.4 Input Kernels for the IOKR Models 3

S.5 Run-time Analysis 5

S.5.1 Setup 5

S.5.2 Results 6

S.6 Analysis of the Marginals 6

S.6.1 Setup 6

S.6.2 Results 6

S.1 Code and Data links

The code and data used in this publication are available at `https://github.com/aalto-ics-kepaco/msms_rt_score_integration`.

S.2 Hyper-parameter Estimation

Algorithm 1 provides the pseudo-code for the hyper-parameter selection procedure, which is verbally described in Section 3.5, to determine the optimal retention order weight D^* given a labeled training set $\mathcal{D}_{train}$. The algorithm can be easily extended to also determine the optimal parameter k^* of a sigmoid function, by searching a grid of $(D, k) \in \mathbf{D} \times \mathbf{k}$ tuples. This latter extension was used to determine (D^*, k^*) for the evaluation of the chain-graph approach (Sec. 3.6 and 4.2) using the Hinge-Sigmoid as edge-potential function.

Algorithm 1: Hyper-parameter Estimation Procedure. This procedure is applied to the sum- as well as max-marginals.

Data: $\mathbf{D}$ retention order weight (D) grid; $\mathcal{D}_{train}$ labeled set of MS-features with candidate set used to evaluate the performance of each grid value.
Result: $D^* \in \mathbf{D}$ retention order weight with the highest performance.

$s \leftarrow \{D : -1 \mid D \in \mathbf{D}\}$;
for $D \in \mathbf{D}$ **do**
  /* Get normalized marginals for tree sample $\mathbb{T}$ (Sec. 2.3). */
  for $t \in \{1, \ldots, L\}$ **do**
    $p(\cdot \mid T_t) \leftarrow$ `get_normalized_marginals`($\mathcal{D}_{train}$, T_t, D);
  /* Get average marginal (Sec. 2.3). */
  $\bar{p}(\cdot) \leftarrow$ `get_averaged_marginal`($\{p(\cdot \mid T_t)\}_{t=1}^{L}$);
  /* Evaluate the performance of D on $\mathcal{D}_{train}$ (Sec. 3.5). */
  $s(D) \leftarrow$ `get_top20AUC_performance`($\bar{p}(\cdot)$, $\mathcal{D}_{train}$);
$D^* \leftarrow \arg\max_{D \in \mathbf{D}} s(D)$;

Table S1: Identification accuracies (top-k) for different edge potential functions. We use the max-marginal and $L = 128$ for the score integration. The accuracies are averaged across all datasets and ionizations. Both potential functions improve significantly ($p < 0.001$, one-sided Wilcoxon signed-rank test) over the *Only MS*^{2} setting.

**Method**	**Edge-potential**	**Top-1**	**Top-5**	**Top-10**	**Top-20**
MS^2 + RT	Sigmoid	21.3	52.9	64.0	74.3
	Step-function	20.8	52.6	64.3	74.4
Only MS^2	-	16.7	49.5	60.4	70.6

S.3 Results

This section contains additional results not shown in the main document.

S.3.1 Comparison of the Edge Potential Functions

In Table S1 we compare the metabolite identification performance of the different edge potential functions presented in Section 2.2. The results are discussed in Section 4.1. The sigmoid function can outperform the step-function significantly for top-1 ($p < 0.001$) and top-5 ($p < 0.05$). As, on the other hand, the improvement of the step- over the sigmoid function is not significant, we decided to use the sigmoid function for the majority of our experiment.

S.3.2 Retention Order Prediction Model

Table S2 shows the retention order prediction accuracy of the RankSVM models for the different evaluation datasets (Sec. 3.1) evaluated on the random subsets used for our score integration experiments. It furthermore compares the RankSVM with the CDK XLogP in terms of their retention order modelling performance. The predicted LogP values are used by MetFrag 2.2 to predicted the candidates' retention times and subsequently re-rank them (Sec. 3.6). The RankSVM scores ($\mathbf{w}^T \phi_{ir}$, Sec. 2.2, *Edge Potential Function*) and the CDK XLogP values, can be considered as a proxy for retention order behavior.

Table S2: The average pairwise prediction accuracy calculated for the correct molecular structures of each subsample. It expresses the agreement of the RankSVM and CDK XLogP with the observed retention orders.

		**Order prediction accuracy**	
**Dataset**	**Ionization**	RankSVM	CDK XLogP
CASMI 2016	negative	0.84 (± 0.04)	0.79 (± 0.05)
	positive	0.83 (± 0.04)	0.72 (± 0.05)
EA (Massbank)	negative	0.87 (± 0.02)	0.81 (± 0.03)
	positive	0.88 (± 0.02)	0.75 (± 0.05)

S.4 Input Kernels for the IOKR Models

This section contains a description of the MS^2 and fragmentation tree (FT) (Böcker and Dührkop, 2016) kernels used for the IOKR models described in Section 3.3 of the main document. FTs are a representation of the fragmentation process a molecule undergoes during the MS^2 analysis. The tree is deduced from the given MS^2 spectrum. Its nodes represent the predicted molecular formulas for each MS^2 peak. The edges are labeled with a predicted molecular formula of the loss between two peaks in the spectrum (Böcker and Rasche, 2008; Böcker and Dührkop, 2016). We used 16 kernels for our models of which one, PPK, is a spectra kernel and 15 are FT kernels. An overview of the kernels can be found in Table S3.

Table S3: Description of the MS^2 and fragmentation tree kernels used for the IOKR models. Read (Dührkop, 2018; Dührkop *et al.*, 2015) for further details. Abbreviations: Molecular formula (MF), Fragmentation tree (FT). The nodes, in a FT, are associated with spectra peaks and the edges are associated with losses.

**Abbreviation**	**Name**	**Description**
CPJXB	Common Path Joined Binary	Number of paths with equal union of losses
CPJ	Common Path Joined	Count of length two paths with the same loss
LC	Loss count	Count of each loss in the FT
LI	Loss Intensity	Intensity weighted counts of common losses
LPC	Loss Pair Count	Count for each pair of consecutive losses in the FT
MLIP	Maximum Loss in Path	Maximum frequency if each loss in any path of the FT
NB	Node Binary	Number of nodes sharing the same molecular formula
NI	Node Intensity	Intensity weighted variant of NB
NSFLC2	Node Loss Interaction	Counts the common paths and weights them by comparing the MF of their terminal fragments
RLB	Root Loss Binary	Number of common root-losses
RLI	Root Loss Intensity	Intensity weighted variant of RLB
UFS1	Substructure in Losses and Leafs	Number of times a predefined set of MFs is preserved in a path or cleaved of intact
UFS3	-	Same as UFS1 but values taken to the power of three
WFPC	Weighted Fingerprint Path	Count paths in the FT that correspond to certain molecular properties
WNSF	Weighted Substructure Counting	Count set molecular substructures present in the training and weight by their occurrence
PPKr	Probability Product Kernel	Probability product kernel computed on the peaks of preprocessed MS^2 spectra

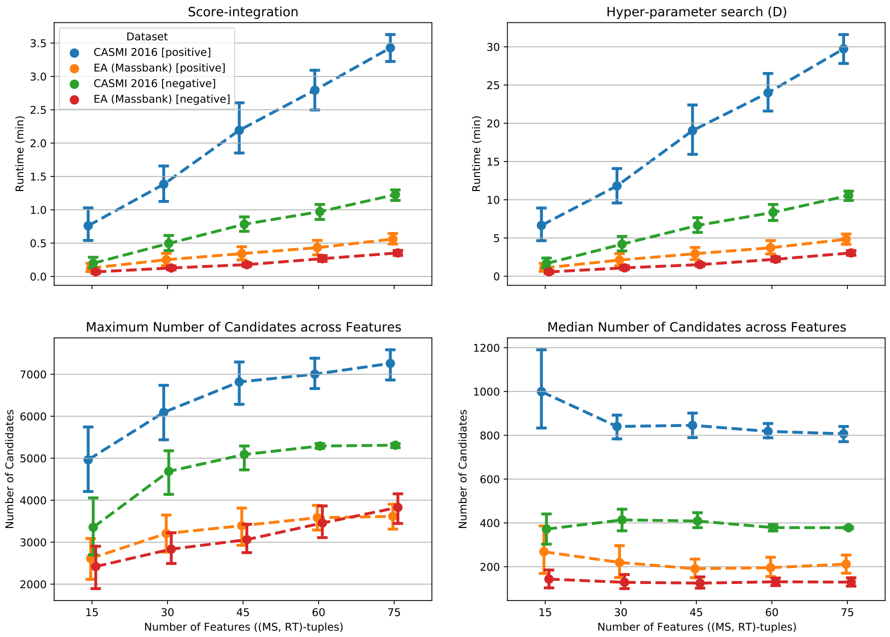

Figure S1: **Upper row:** Run-times for the scoring- and training-phase for different number of MS-features. **Lower row:** Maximum and median number of candidates in the MS-feature samples. We plot the curves separately for the different evaluation sets used in the main paper. Error bars indicate the 95% confidence interval determined using bootstrapping. The score-integration was run using the sigmoid edge-potential function using the max-marginal and $T = 128$ random spanning trees as parameters.

S.5 Run-time Analysis

S.5.1 Setup

We measured the run-time of our score-integration framework on a computer equipped with a Intel® Xeon® Processor E5-2680 v3 CPU (12 cores) and 128GB RAM. We calculate the marginals of the different random spanning trees in parallel on the different cores of the CPU. The reported run-times neither include the time needed calculate the MS^2 candidate scores, e.g. using MetFrag, nor the calculation of the preference scores using the RankSVM (see Section S.3.2). The RankSVM preference scores, however, only need to be computed ones for a molecular candidate set database.

As stated in Section 2.3 (Run-time Complexity) the run-time depends on the number of mass spectrometry (MS) features N, i.e. (MS^2, RT)-tuples, and the (maximum) candidate set size assigned to the MS-features. In practice, those parameter very depending on the analysed data. For example, in our experiments the median number of candidates for an MS-features lays between 119 and 919 depending on the evaluation dataset (see Table 1). Therefore, we measured the run-time for each evaluation dataset separately and also varied the number of MS-features ($N \in \{15, 30, 45, 60, 75\}$). For each (dataset, N) combination we repeated the run-time measurement 15 times on a randomly selected MS-feature subsample. We differentiate between the training phase of our framework, i.e. determining the optimal D (typically done only ones, compare Section 3.5 and S.2), and scoring phase (repeatedly applied to new datasets).

Table S4: Maximum memory requirement throughout the run-time experiments.

		Maximum Memory (GB)	
**Dataset**	**Ionization**	**Per CPU-Core**	**Total**
EA (Massbank)	Positive	0.75	9.0
EA (Massbank)	Negative	0.46	5.5
CASMI 2016	Positive	2.7	32.0
CASMI 2016	Negative	1.11	13.3

Table S5: Statistics of the marginal-score distribution based on all MS-features used in our evaluation. We differentiate between score for correct and incorrect highest ranked (top-1) candidates.

Top-1 Correct	Minimum	Median
False	0.80	0.98
True	0.89	1.00

S.5.2 Results

Figure S1 (upper row) shows the run-time for the score-integration (application phase) and the hyper-parameter search (training phase) as function of the number of MS-features (N) for the different datasets. It can be seen, that the run-time grows approximately linear with N in both phases. However, there is significant difference between the datasets which can be explained by the number of candidates (see Figure S1, lower row). One can see, that the higher the number of maximum number of candidates the longer the run-time is. However, we also see that the run-times are suitable for practical applications. For example, re-ranking $N = 75$ (MS^2, RT)-tuples with a maximum number of candidates around 5200 takes about 80sec. Determining the optimal retention order weight D, for the same setting, runs in about 11min.

Table S4 shows the maximum memory used during the run-time experiments. Again, we see that the larger the candidate sets are the larger the memory consumption is. However, the required memory stays within the boundaries of modern workstations.

S.6 Analysis of the Marginals

S.6.1 Setup

We analysed the averaged max-marginal scores (see Equations (4) and (5), here denoted simply with marginal-scores) for the highest ranked candidate (top-1) structures of all (MS^2, RT)-tuples used in our evaluations (see Section 3.1). All marginal-scores where calculated using 128 random spanning trees, sigmoid edge-potential function and MetFrag was used as MS^2-Scorer (compare "MS^2 + RT (*our*)" in Table 2).

S.6.2 Results

In Figure S2 we show the empirical cumulative distribution function (ECDF) of the if the marginal-scores. It can be seen, that the marginal-scores tend to be higher for true top-1 structures than for the false ones. As we are using max-marginals (compare Eq. (5)), this indicates that for correct top-1 structures the random spanning tree ensemble tend to agree more, i.e. resulting in the same highest ranked structure for most trees. On the opposite, if the highest ranked structure is incorrect the tree ensemble seems to output a more diverse set of top-1 structures resulting in lower averaged max-marginal scores (compare Eq. (4)) from the tree ensemble.

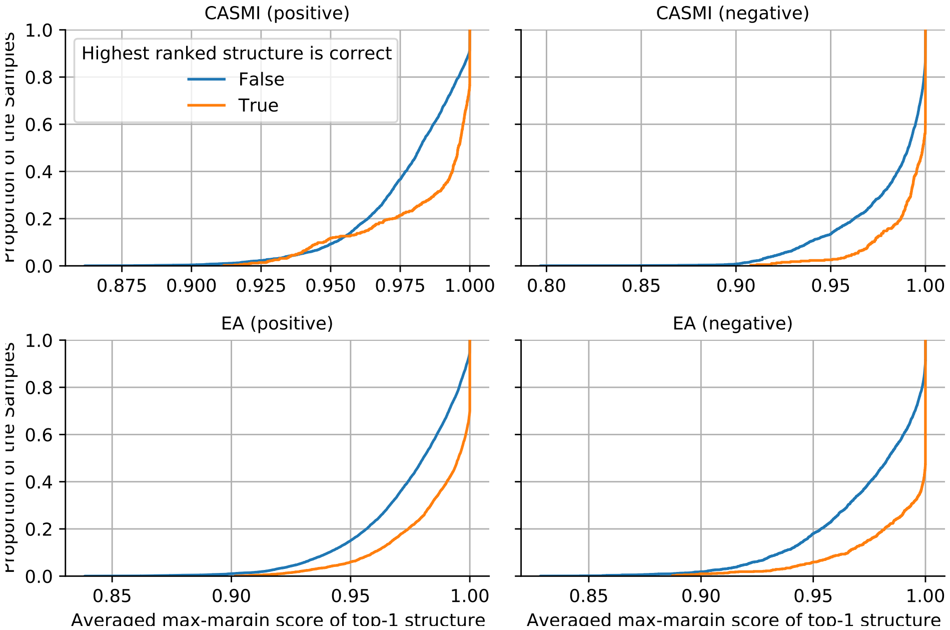

Figure S2: Empirical cumulative distribution functions (ECDF) of the marginal-scores for the top-1 candidate structures of the MS-features in our evaluation sets. We plot two ECDFs for each (dataset, ionization) combination, differentiating between correct and incorrect top-1 molecular structures.

Table S5 shows statistics of the marginal score distribution from the pooled samples of all datasets and ionizations. The median marginal-score value significantly differs between the correct and incorrect top-1 structures (Kruskal-Wallis H-Test, $p = 0.0$). This indicates that for correct top-1 structures the marginal-scores tend to be higher.

In Figure S3 we analysis how the correct and incorrect top-1 structures are distributed for different marginal-score ranges, i.e. if τ denotes the marginal-score than either $\tau \in (r_{\text{low}}, r_{\text{high}}]$ (distribution) or $\tau \in (-\infty, r_{\text{high}}]$ (cumulative distribution)). From the empirical distribution (left bar-plot of Figure S3) we can see the tendency that lower marginal-scores result in lower true-positive (TP) rates. For example, for a top-1 candidates with a marginal-score close to 1.0 we have a TP rate of about 40%, whereas the TP rates for scores around 0.95 already drops to around 11%. That means, if our score-integration framework ranks a molecular structure at top-1 with a high score (close to 1.0) we can be more confident that this structure is correct, than if its marginal-score is lower. From the cumulative distribution (right bar-plot in Figure S3) we can also clearly see the jump in the TP rate for top-1 marginal-scores close to one.

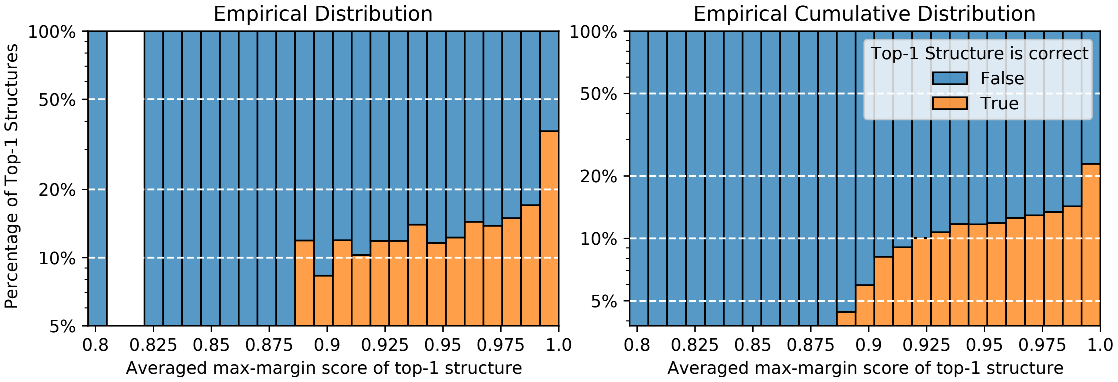

Figure S3: Empirical distribution of correct and incorrect top-1 structures are different marginal-score ranges. **Left:** Histogram with a bin-width of about 0.0075. **Right:** Cumulative histogram.

References

Böcker, S. and Dührkop, K. (2016). Fragmentation trees reloaded. *Journal of Cheminformatics*, **8**(1), 5.

Böcker, S. and Rasche, F. (2008). Towards de novo identification of metabolites by analyzing tandem mass spectra. *Bioinfomatics*, **24**(16), i49–i55.

Dührkop, K. (2018). *Computational Methods for Small Molecule Identification*. Ph.D. thesis, Friedrich-Schiller-Universität Jena.

Dührkop, K., Shen, H., Meusel, M., Rousu, J., and Böcker, S. (2015). Searching molecular structure databases with tandem mass spectra using CSI:FingerID. *Proceedings of the National Academy of Sciences*, **112**(41), 12580–12585.
